# Proton radiography and tomography with application to proton therapy

**DOI:** 10.1259/bjr.20150134

**Published:** 2015-07-01

**Authors:** G Poludniowski, N M Allinson, P M Evans

**Affiliations:** ^1^ Centre for Vision Speech and Signal Processing, Faculty of Engineering and Physical Sciences, University of Surrey, Guildford, UK; ^2^ Department of Medical Physics, Karolinska University Hospital, Stockholm, Sweden; ^3^ Laboratory of Vision Engineering, School of Computer Science, University of Lincoln, Brayford Pool, Lincoln, UK

## Abstract

Proton radiography and tomography have long promised benefit for proton therapy. Their first suggestion was in the early 1960s and the first published proton radiographs and CT images appeared in the late 1960s and 1970s, respectively. More than just providing anatomical images, proton transmission imaging provides the potential for the more accurate estimation of stopping-power ratio inside a patient and hence improved treatment planning and verification. With the recent explosion in growth of clinical proton therapy facilities, the time is perhaps ripe for the imaging modality to come to the fore. Yet many technical challenges remain to be solved before proton CT scanners become commonplace in the clinic. Research and development in this field is currently more active than at any time with several prototype designs emerging. This review introduces the principles of proton radiography and tomography, their historical developments, the raft of modern prototype systems and the primary design issues.

Despite a history going back over 50 years,^1^ proton radiography (pRG) and tomography have been slow to reach the clinic.^2^ Few manufacturers currently offer a clinical imaging system suitable for pRG and none for proton tomography. In fact, it turns out that the use of protons instead of X-rays for transmission imaging has some disadvantages. These include the need for large expensive equipment to produce proton beams (*e.g.* a cyclotron or synchrotron) and the limitations on image quality arising from the multiple scattering of protons.

Proton sources of sufficient energy do, however, exist for several purposes, one application being for proton therapy. The multiple scattering effects remain a fundamental difficulty: protons do not move through a medium in straight lines. So why should we even attempt proton transmission imaging? The prime motivation is with application to proton therapy planning. It was Cormack^1^ who was the first to realize the possibilities of proton CT (pCT). In a seminal article of the 1960s on tomographic reconstruction, the Nobel Laureate wrote:

The next application of the solution [for CT] … concerns the recent use of the peak in the Bragg curve for the ionization caused by protons, to produce small regions of high ionization in tissue. The radiotherapist is confronted with the problem of determining the energy of the incident protons necessary to produce the high ionization at just the right place, and this requires knowing the variable-specific ionization of the tissue through which the protons must pass.

This is still a fair assessment of the problem facing any proton therapy team today. Cormack went on to propose that the energy loss of protons passing through a patient can tell us about proton stopping power inside the patient—something that X-rays can never give us directly.

Typically, in both photon and proton external beam therapy, prior to treatment, an X-ray CT scan is acquired for treatment planning purposes. This is used for outlining structures, but also provides a map of electron density that is used to calculate dose deposition. In proton therapy, the translation of electron density to proton stopping power provides an extra and appreciable source of error. The most advanced X-ray CT calibration method in common usage is probably the stoichiometric method.^3^ The resulting overall uncertainty (1*σ*) in stopping-power ratio (SPR) for protons in different tissue types has been estimated as 1.6% (soft tissue), 2.4% (bone) and 5.0% (lung).^4^ As an illustration, note that the estimate of 1.6% for soft tissue includes contributions for (added in quadrature): stoichiometric parameterization (0.8%), human tissue composition variation (1.2%) and mean excitation energy (0.2%) and other sources (0.6%). None of the first three sources of errors contribute in a calibration in pCT and the ambition with this type of imaging should be to reduce the uncertainty in SPR substantially (to <1%). Reduced uncertainties offer the possibility of smaller planning margins and additional beam directions, potentially leading to superior patient outcomes. The surge in the number of operational and planned proton therapy centres in recent years therefore makes the exploitation of this modality timely.^5^

Before proceeding further, some clarification of topic coverage should be made. pRG and pCT, in the context of this review, mean the imaging of an object using the transmission of protons through it. The energy loss of the transmitted protons is the primary mechanism for image contrast. The greatest emphasis will be given to proton-tracking systems: as will be seen, these are best able to cope with the difficulties imposed by proton multiple scattering. Some requirements for a practical pCT scanner for proton therapy are summarized in Table 1. Note that the dose burden expected from this form of imaging is not unduly high. The estimated absorbed dose required for a pCT scan of a head, for treatment planning purposes, has been estimated at a few milligray.^10^ For comparison, note that a typical head scan using a diagnostic X-ray CT scanner or X-ray cone beam CT (CBCT) might deliver 40 mGy.^11^

**Table 1.** Requirements for a practical (proton-tracking) CT scanner for proton therapy

Category	Parameter	Value
Proton beam	Energy	≥200 MeV (head)
		≥250 MeV (body)
	Flux*^a^*	≥3000 protons cm^−2^ s^−2^
Imaging dose	Maximum absorbed dose*^b^*	<20 mGy
Image quality	Spatial resolution, *σ*	≈1 mm
	Relative stopping-power accuracy	<1%
Time	Data acquisition time	<10 min
	Reconstruction time	<10 min

^*a*^ Quoted figure based on the scenario of 1-mm voxels and 180 projections, a target of 100 protons passing through a voxel per projection^6^ and a 10-min acquisition.

^*b*^ Quoted figure based on a crude calculation of comparable stochastic risk to typical X-ray CT head scans (≈40 mGy^7,8^), assuming a proton radiation weighting factor twice that of photons.^9^

We will not be concerned here with other forms of imaging using proton beams, such as nuclear scattering tomography^12^ that relies on wide-angle scattering, γ interaction vertex imaging^13^ (GIVI) using prompt γ emission or positron emission tomography^14^ (PET) of induced β emission. The latter two (GIVI and PET) primarily promise benefit for *in vivo* range verification (inferring the depths that protons penetrated).^15^ Finally, we emphasize that our interest in this review is with protons. Reference to heavy-ion radiography and tomography will be made only where comparison with imaging with protons is apt, and we refer the reader to other sources^16^ for this related topic.

## AN OVERVIEW OF THE PHYSICS OF PROTON IMAGING

Typically protons lose their energy gradually as they penetrate into a material and the rate of energy loss increases as they slow down, producing a sharp “Bragg peak” at their terminus. The stopping depth is quite well defined for a particular initial energy. Proton therapy takes advantage of this characteristic to concentrate a high dose in a tumour with very little dose deposited beyond the proton range. Typical initial kinetic energies for therapeutic applications extend from around 60 MeV (3 cm range in water) to 230 MeV (33 cm range in water). Henceforth, when the term proton energy is used in this review, it should be taken to refer to its kinetic energy.

Any therapeutic energy proton passing through an appreciable thickness of tissue (>1 mm water) will undergo many interactions. Owing to the stochastic nature of charged particle interactions, there will be statistical variations in:^17^

(i) lateral position at a given penetration depth (“lateral straggling”)
(ii) proton direction at a given penetration depth (“angular straggling”)
(iii) energy at a given depth (“energy straggling”)
(iv) stopping depth for a given initial energy (“range straggling”).

Representative numbers for these phenomena are provided in Table 2. Given the statistics for lateral straggling, obtaining the target spatial resolution listed in Table 1 is clearly a challenge.

**Table 2.** Illustrative statistics for proton straggling effects (200-MeV protons)

Depth (cm)	200-MeV proton incident on water				
	*σ*_x_ (cm)	*σ*_θ_ (mrad)	*σ*_E_ (MeV)	*E*_m_ (MeV)	*σ*_R_ (cm)
5	0.04	15	0.8	176.6	–
10	0.11	20	1.2	150.9	–
20	0.37	41	2.2	86.3	–
At range	–	–	–		0.29

*σ*_x_, spatial straggling (arbitrary lateral dimension); *σ*_θ_, angular straggling (arbitrary lateral direction); *σ*_E_, energy straggling; *σ*_R,_ range straggling; *E*_m_, mean proton energy at depth.

Figures are based on simulations by the authors using the FLUKA Monte Carlo code.^18^ Gaussian fits were used to determine *σ*_x_, *σ*_θ_ and *σ*_R_ and root mean square deviation to determine *σ*_E_.

The random deviations in proton direction are predominantly caused by elastic Coulomb scattering from the nuclei of atoms: so-called “multiple Coulomb scattering” (MCS). This in turn produces lateral deviations and the two forms of straggling are correlated. Energy loss and its variation, however, are predominantly caused by excitation and ionization of atomic electrons: this is described by the “Bethe formula” and its extensions. The stopping depth for any particular proton exhibits statistical variation owing to variations in cumulative energy loss, although variations in non-linear paths also contribute to a lesser degree. Range straggling is therefore intimately connected with energy straggling. The standard deviation in range straggling typically slightly exceeds 1% of the range.^19^

In addition to these processes, rarely, at a rate of approximately 1% per cm at therapeutic energies, a proton may undergo any of an array of inelastic nuclear interactions, including absorption. Such catastrophic nuclear interactions can be considered to remove the proton from the beam and to reduce the primary fluence.^17^

Proton therapy requires that the protons stop in the vicinity of the tumour. Proton transmission imaging, however, requires that the protons pass through the patient and reach a detector. This latter aim is achieved by increasing the initial energy above that required for therapy. The energy loss of each proton is the primary mechanism for generating image contrast. This is unlike radiography, which has traditionally relied primarily on the reduction in fluence in a primary beam. If the aim of pRG is an estimate of stopping power within the patient rather than purely anatomical imaging, we face an apparent problem. By increasing the initial proton energy for imaging, measurements of stopping power are made at an inappropriate energy for therapy. However, SPR, that is, the ratio of stopping power at a point relative to that for water, is approximately constant with energy and its slow variation is well understood.^3^ It is this fact that makes proton transmission imaging potentially so useful for treatment planning.

The goal of pRG/pCT data acquisition is to arrive at a set of values of water-equivalent path lengths (WEPLs) through the patient. Each WEPL value is a line-integral of SPR and analogous to a ray-projection in radiography. WEPL can be determined in a number of ways. A calibration can be made between the signal in a detector and the path length traversed, averaged over many protons: these systems will be referred to as proton integrating. In another approach, measurements can be made of each proton's residual energy or range after emerging from the patient: such systems will be referred to as proton tracking. In pRG, two-dimensional (2D) images of mean WEPL may be used for the verification or correction of X-ray planning CT scans. An image of uncertainty in WEPL (related to the “range dilution”) can additionally be obtained with tracking systems and this also has potential benefit for planning.^2^ In pCT, a final reconstruction step is carried out to obtain SPR in a three-dimensional volume from the WEPL measurements.

Regardless of detector technology, image quality in pRG/pCT is impacted by straggling effects within the patient. Energy-range straggling is a form of noise that can be suppressed by increasing the number of protons used for imaging. Lateral straggling, however, limits the obtainable spatial resolution and may also result in image artefacts. It can be suppressed by raising the initial proton energy further, but that is achieved at the cost of reduced energy contrast through the patient. It should be noted that heavier ions exhibit lesser lateral straggling than protons and therefore transmission imaging for heavy-ion therapy is, in some sense, a simpler problem.

## HISTORICAL SURVEY

**Figure 1.**
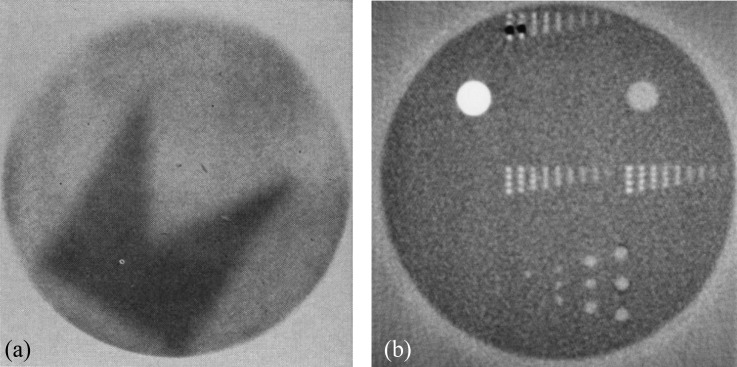
(a) The first published proton radiograph from 1968.^20^ Reprinted from Koehler^20^ with permission from The American Association for the Advancement of Science. (b) A slice image of a 29-cm diameter phantom from the Los Alamos proton CT scanner in 1978.^21^ © 1978 IEEE. Reprinted from Hanson et al^21^ with permission from IEEE.

The first examples of pRG were demonstrated in the 1960s. Although the instrumentation to perform the measurements was not new in 1968, Koehler was probably the first to publish a planar radiograph. For the exposure at the Harvard Cyclotron (Cambridge, MA), a proton beam was spread by scattering and directed on to photographic film.^20^ The film was placed close to the proton range and use was made of the sharp drop in proton fluence at this location. This first image is reproduced in Figure 1a where the contrast is generated by the addition of a 100-μm pennant-shaped sheet of aluminium. Other proton radiographic works followed^22,23^ but the use of fluence as the mechanism for contrast limited the application of projection radiography to thin samples.

Tomographic reconstructions of Goitein,^24^ based on data acquired by Lyman, deserve a mention, although this was α-particle transmission imaging. In 1972, Goitein reconstructed CT images using data from the α-beam of the Lawrence Berkeley Laboratory Cyclotron (Berkeley, CA) and an iterative reconstruction algorithm. This is the first example of transmission tomography using a charged particle and also of the energy loss of individual particles being utilized for contrast. An α-particle scanner was developed at the same laboratory and even trialled on humans.^25^

The first charged particle CT reconstruction using protons seems to have been published in the mid 1970s, appropriately enough, by Cormack and Koehler.^26^ For a narrowly collimated 158-MeV pencil beam, the WEPL for paths through a phantom were inferred using NaI scintillators coupled to photomultiplier tubes. This was a proton-integrating system where individual protons were not tracked. The reconstruction was performed analytically using Abel's equation and the property that the phantom was circularly symmetric. No reconstructed image was included in the publication, although a line-profile was presented.

Hanson et al^21^ at the Los Alamos Laboratory (Los Alamos, NM), took up the development of pCT in the late 1970s and early 1980s, with a series of articles that culminated in the scanning of human specimens.^21,27–29^ The first pCT images that the authors of this review have identified were published by this group. An early image is reproduced in Figure 1b. A 240-MeV proton pencil beam was used for imaging and the phantom consisted of a plastic cylinder with inserts of varying size and density. Two varieties of detector module with very different functions were utilized: a position-sensitive detector (PSD) and a residual energy-range detector (RERD). These concepts are still relevant for the design of proton imaging systems today. The former tracks each proton's position and the latter implies its residual energy or range. In the Los Alamos system, a multiwire proportional chamber was used as a PSD, determining the proton exit position at a plane downstream of the phantom. In the early experiments, a hyperpure germanium detector was used as a RERD to determine residual energy (a “calorimeter”). In later experiments, a stack of plastic scintillators was used to determine proton stopping depth (a “range telescope”). The Los Alamos work was a huge step forward, both conceptually and experimentally. The ideas of determining proton exit angle and applying cuts to the proton exit trajectory were suggested to improve spatial resolution.^27^ The possibility of using curved projection paths was also discussed. A proton rate in excess of 10 kHz was obtained with a version of the system.^29^ Hanson^27^ considered future developments:

In the present discussion, we will concentrate on the feasibility of scanning a patient in 10 s with a proton beam. The objective would be to accumulate 10^8^ events with which to make a CT reconstruction … At first sight the data handling problems associated with a 10 MHz data rate appear formidable. However, upon closer inspection, these problems are found to be soluble with present-day technology with only a modest amount of multiplexing and parallel processing.

This statement was made back in 1979 and proved somewhat optimistic given that, as we shall see, developers are still struggling to realize a 10-MHz proton rate in modern prototype systems.

At the start of the 1980s, the major technological and conceptual elements were all in place to enable the development and deployment of proton radiographic and tomographic systems in the clinic. With few exceptions,^30,31^ little attention was given towards this goal in the next decade and a half. It is possible to view this lull as a pause between proof-of-principle and timeliness for exploitation. In this review, the modern era of pRG and tomography is considered to commence with the systems developed at the Paul Scherrer Institute (PSI) (Villigen, Switzerland) from the mid 1990s.^32,33^ The modern era is characterized by a strong focus on the application of pRG/pCT to range verification and treatment planning in proton therapy.

## THE MODERN ERA

### Proton-integrating systems

**Figure 2.**
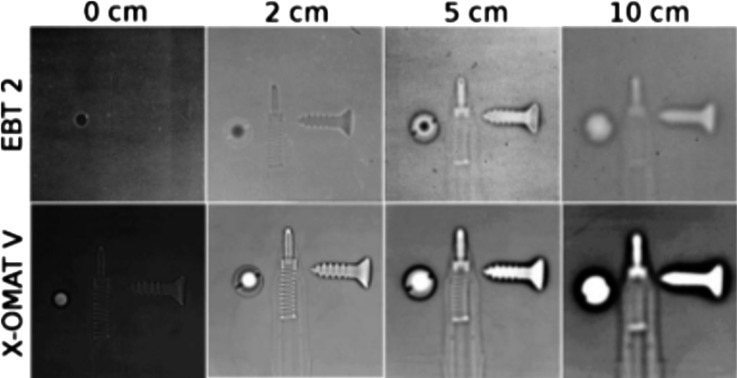
Radiographs of a pen tip and screw acquired with radiochromic film and varying air gap offsets, published in 2011.^34^ Images with two types of radiochromic film are presented: with EBT 2 (Ashland Inc., Covington, KY) and with X-OMATV (Carestream Health, Rochester, NY). Reprinted from Seco and Depauw^34^ with permission from the American Association of Physicists in Medicine.

Before discussing the most advanced modern pRG/pCT systems using proton tracking, developments with proton-integrating technologies will be summarized. A proton transmission radiograph can be obtained by directing a proton beam through an object and on to a suitable sensor. The passage of protons is detected indirectly, typically exploiting its transfer of energy *via* ionization and excitation. The definition of proton-integrating technology is that signal (*e.g.* in a pixel) is due to the passage of an undetermined number of incident protons. The resulting signal will depend on both proton fluence and energy distribution, but proton-integrating radiography assumes that the signal can be calibrated to average proton WEPL through the patient. The limitations of the proton-integrating approach are illustrated in Figure 2. Radiographs were acquired of a pen tip and a screw with varying air gaps using radiochromic film and a 117-MeV proton beam.^34^ The interplay of MCS and energy loss results in a “halo” effect at material interfaces which increases with receptor offset. The degradation in spatial resolution for integrating compared with tracking systems will depend on the patient anatomy and the detector–patient geometry. A variety of detector technologies have been demonstrated in the context of proton-integrating radiography.

**Figure 3.**
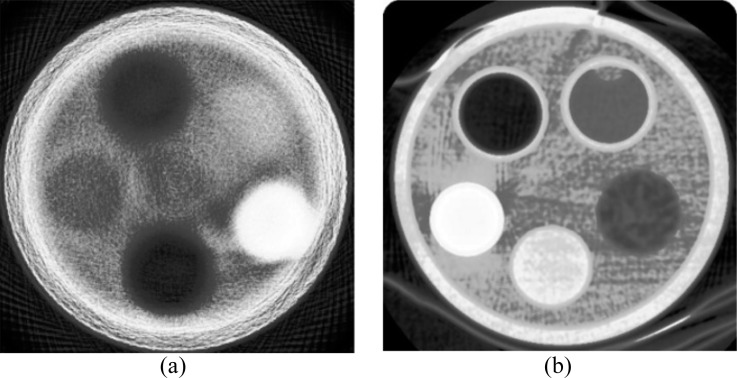
(a) Slice image from the Harvard Cyclotron proton CT scanner published in 2000 and (b) a slice image from a contemporary X-ray CT scanner (GE 9800).^35^ The phantom diameter is 9.5 cm. © Institute of Physics and Engineering in Medicine. Reproduced from Zygmanski et al^35^ with permission from IOP Publishing. All rights reserved.

At the turn of the millennium, pCT was demonstrated using a 159-MeV proton beam at the Harvard Cyclotron.^35^ A gadolinium oxysulfide scintillator screen was coupled to a charge-coupled device (CCD) and the signal calibrated to WEPL. Tomographic reconstruction was performed using the Felkamp algorithm: a filtered backprojection (FBP) method commonly used with X-ray CBCT systems.^36^ An attempt was made to correct projections for scattering effects prior to reconstruction. Recognizable phantom images were obtained, but severe edge artefacts were still present at interfaces between materials owing to the MCS. This is illustrated by a phantom slice image in Figure 3a in comparison to that of a then contemporary X-ray CT scanner (Figure 3b). The same scintillator-CCD approach has been explored by other groups.^37^ The same system concept has been applied to heavy ion CT, where MCS effects are typically lower, resulting in superior image quality.^38^

Flat-panel detector arrays based on amorphous silicon technology have become commonplace in the past decade for image guidance in photon therapy. It is unsurprising therefore that this is a candidate technology for pCT. The principle has been demonstrated for carbon-ion radiography with a commercial flat-panel device: a gadolinium oxysulfide scintillator coupled to an amorphous silicon matrix array.^39^ An FBP approach was used for reconstruction and showed impressive results: an SPR accuracy of 1% and spatial resolution dominated by the pixel size (0.8 mm). It seems inevitable that a similar setup will be attempted for pCT, although the increased MCS of protons with respect to carbon ions will lead to decreased image quality.

**Figure 4.**
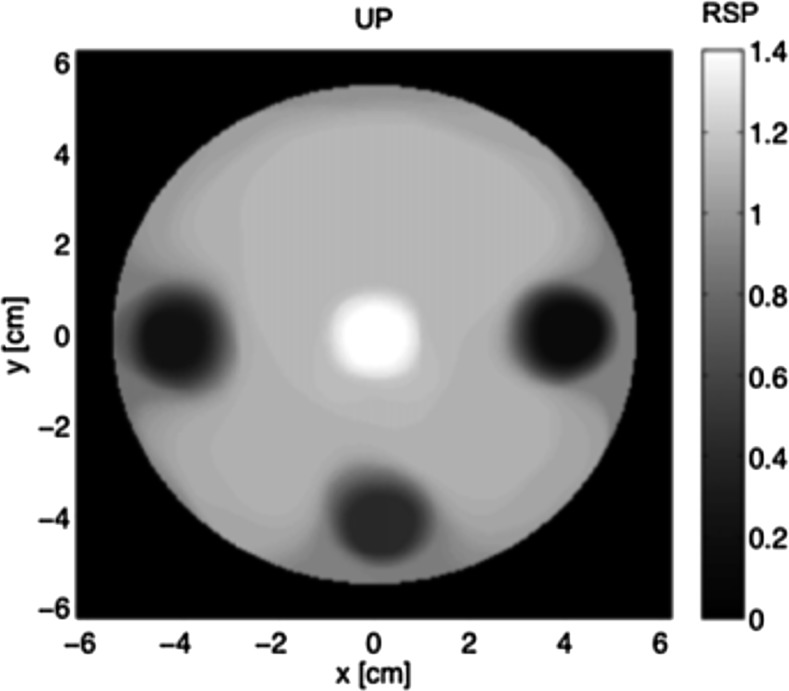
Slice image from the Massachusetts General Hospital proton CT scanner published in 2013.^40^ The phantom diameter is 12 cm. RSP, relative stopping power, i.e. stopping-power ratio. © Institute of Physics and Engineering in Medicine. Reproduced from Testa et al^40^ with permission from IOP Publishing. All rights reserved.

Recently, pCT was demonstrated at Massachusetts General Hospital (Boston, MA) using a clinical proton beam of 175 MeV and a prototype 2D diode-array detector (Sun Nuclear Corporation, Melbourne, FL).^40^ This detector had a 12-cm field size and contained 249 semi-conductor diodes in an octagonal array with a 7-mm diagonal pitch. Reconstruction was by iterative methods. The innovative system provided recognizable CT phantom images, as illustrated in Figure 4. However, the sparseness of the detector array resulted in very low spatial resolution and makes an evaluation of the severity of MCS effects difficult. The errors in reconstructed SPR, in this initial demonstration, were also several times greater than would be acceptable for therapy planning.

The use of complementary metal oxide semi-conductor active pixel sensors (CMOS APSs) has also been explored recently.^34,41^ Proton-integrating projection radiographs of phantoms have been obtained and the suitability of the technology demonstrated. We note that silicon pixel detectors also have potential application in proton-tracking radiography, if the noise level in the sensor can be kept low enough and the frame-rate high enough to resolve individual proton events.^41^

### Proton-tracking systems

**Figure 5.**
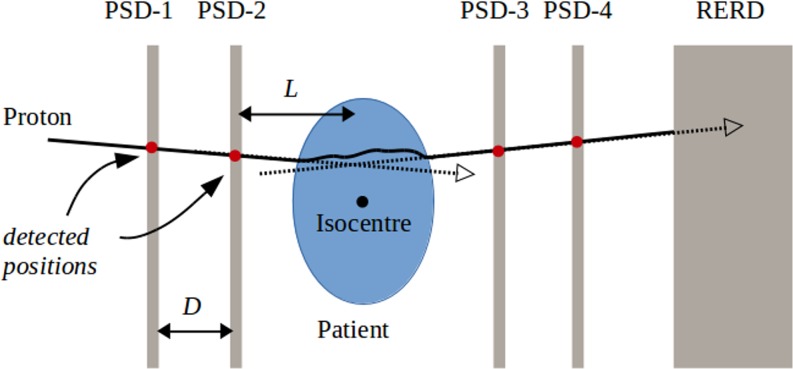
A schematic of the ideal proton-tracking proton radiography/proton CT system. PSD, position-sensitive detector; RERD, residual energy-range detector.

By contrast to proton-integrating devices, proton-tracking radiography and tomography systems consist of a number of PSD modules to infer proton path (typically between one and four) and a RERD to determine its residual energy. This is illustrated in Figure 5. Note that a detector to measure initial proton energy would also be advantageous, although no suitable detector has yet been proposed as a part of any prototype system. A precise determination of proton energy would be required (<1 MeV) without substantially perturbing the proton's path or degrading its energy. A summary of the pRG/pCT systems that have recently been in development or testing is provided below.

**Figure 6.**
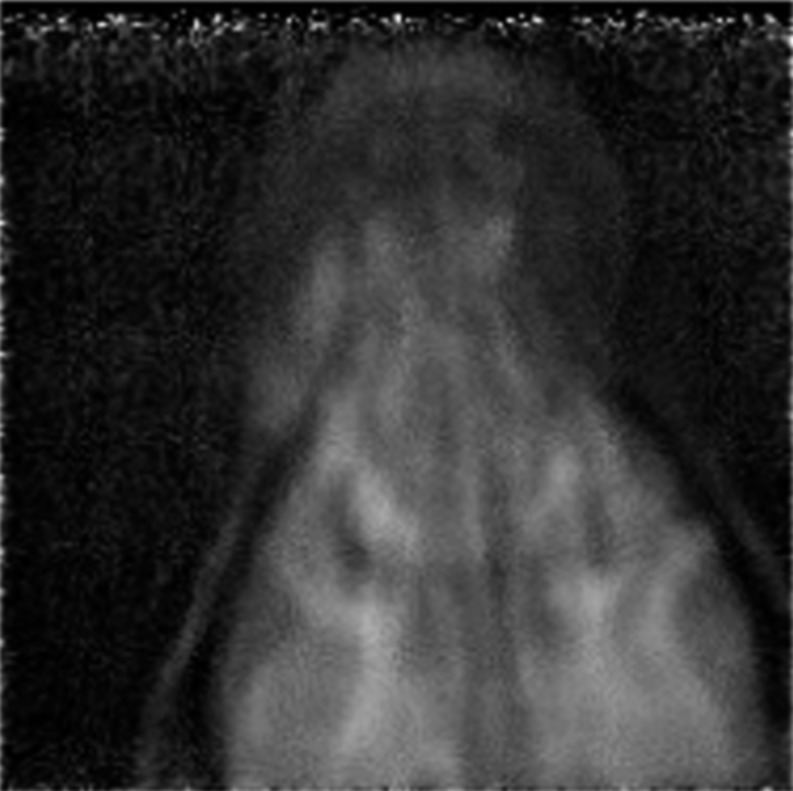
A proton radiograph of a canine's head obtained with the Paul Scherrer Institute system, published in 2004.^2^ Reproduced from Schneider et al^2^ with permission from the American Association of Physicists in Medicine.

In the 1990s, a collaboration centred around the PSI worked towards pRG, culminating in the system described in 1999.^33^ The proton-tracking system consisted of two PSDs (one before and one after the patient). The tracking units were scintillating fibre hodoscopes (Sci-Fis) consisting of two orthogonal planes of 2 × 2 mm^2^ plastic fibres. The fibres were made of plastic scintillator (Bicron BCF 12; decay time, 3.2 ns)^42^ and were each coupled to a channel of a photomultiplier tube. The RERD was a range telescope consisting of 64 closely packed and optically isolated scintillator tiles of 3-mm thickness. The tiles were also made from plastic (Bicron BC404; decay time, 1.8 ns)^42^ and the light from each tile was collected by a wavelength-shifting fibre coupled to a photomultiplier channel. The purpose of the fibres was to collect scintillator emissions and efficiently transfer light quanta to the photon sensor at a wavelength matched to the spectral sensitivity. The PSI pRG system could image a 22.0 × 3.2 cm^2^ area and event rates of 1 MHz were obtained. Experimental planar images were synthesized by the scanning of a pencil beam. Although the system would have been suitable for pCT, there is no indication that the system was ever used for this. However, pRG with a live canine subject was presented^2,43^ and such a radiograph is reproduced in Figure 6.

Between 2003 and 2013, a collaboration including Loma Linda University (LLU), University of California Santa Cruz (UCSC) and Northern Illinois University (NIU) published many important articles on pCT and the development of their prototype system.^6,10,44–53^ In 2010, their prototype was completed, and the first results were presented. The tracking system consisted of four PSDs: two before the patient and two after. This allowed the determination of incoming and outgoing proton direction as well as position. Each PSD consisted of two silicon strip detectors (SiSDs) arranged orthogonally to provide proton *x*-*y* position. Each SiSD had a sensitive area of approximately 9.0 × 9.0 cm^2^ (pitch, 228 μm; thickness, 400 μm). To obtain a larger field-of-view (9.0 × 17.4 cm^2^), the number of SiSDs was doubled. The RERD was calorimeter based and consisted of 18 CsI : Tl crystals (each: 3.5 × 3.5 × 12.5 cm^3^) arranged in a 3 × 6 matrix. The light was collected by a photodiode paired to each crystal. The maximum proton rate obtained with the system was low (10–20 kHz), which led to a CT scan time of several hours. The relatively low rate can be attributed to the dead-time of the calorimeter (decay time, 800 ns)^54^ and the lack of a fast data acquisition system (DAQ). However, protons up to 200 MeV in energy could be imaged (limited by calorimeter thickness) and the accuracy of SPR in the resulting CT images was encouraging (to <1%).^53^ The reconstruction used an advanced iterative method incorporating proton “most likely paths” (MLPs).^51^

**Figure 7.**
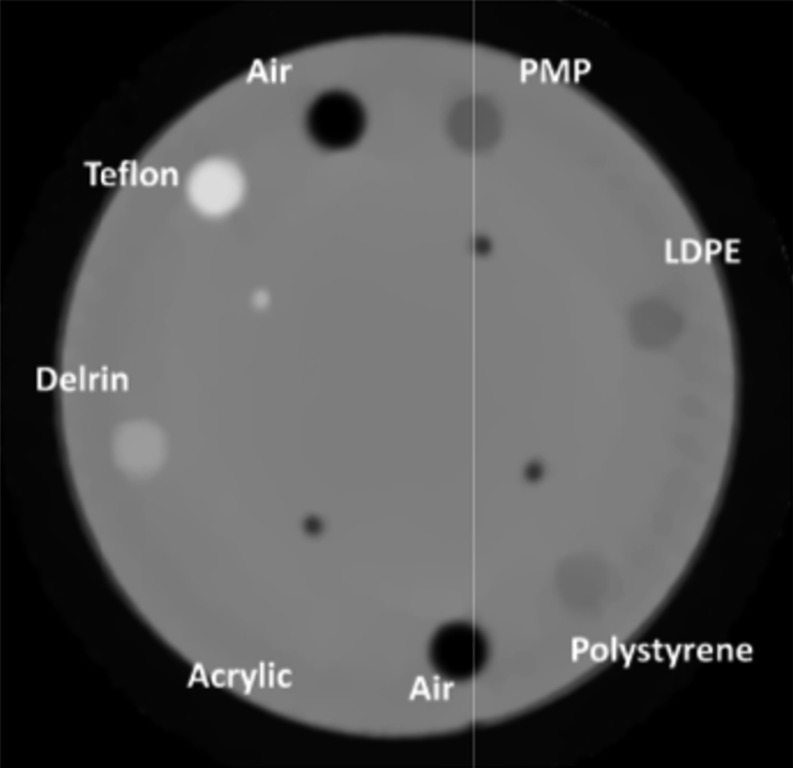
A proton CT (pCT) slice of a Catphan phantom (The Phantom Laboratory, Greenwich, NY) obtained with the Loma Linda University (LLU)/University of California Santa Cruz (UCSC)/California State University, San Bernadino (CSUSB) pCT system. The phantom diameter is 15 cm. LDPE, low-density polyethylene; PMP, polymethyl pentene. Image kindly provided by Robert P Johnson and reproduced with permission of the LLU/UCSC/CSUSB collaboration.

In 2011, LLU, UCSC and California State University, San Bernadino (CSUSB), obtained funding to build a second generation system. The system is again a head scanner capable of imaging proton of energy up to 200 MeV.^55^ The proton-tracking system again utilizes four PSDs consisting of SiSDs and is identical in essential characteristics to the first generation system. The residual range, however, is inferred using a hybrid RERD. This consists of a stack of five fast plastic scintillators read out by photomultiplier tubes. This design provides a more precise determination of residual range, compared with the calorimeter of the first generation system. The DAQ was also upgraded with a design specification of 2 MHz. A proton rate in excess of 1 MHz has already been confirmed experimentally. Early results suggest good SPR accuracy and impressive image quality.^55^ A reconstructed slice of a Catphan phantom (The Phantom Laboratory, NY) is reproduced in Figure 7. The image quality obtained has set a standard that will be a benchmark for other prototypes systems.

In 2008, the Tera Foundation (Novara, Italy) obtained funding from the Italian National Centre for Oncological Hadron Therapy (CNAO) (Pavia, Italy) to develop a series of devices for a project entitled Advanced Quality Assurance in Hadron Therapy.^56^ Proton range radiography was one of the stated objectives and this led to the construction of their PRR30 system.^57–59^ The full-scale system was demonstrated using X-ray beams in 2013. The primary goal of the project was radiography rather than tomography and we are not aware of any use of the PRR30 as a CT acquisition system. The tracking system consisted of two PSDs after the patient, allowing inference of only outgoing proton direction and position. The technology for the trackers was based on three-foil gas electron multipliers (GEMs) with a read out pitch of 400 μm. The RERD was a stack of 48 plastic scintillators (BC-408; decay time, 2.1 ns)^42^ with an area of 30 × 30 cm^2^ and a tile thickness of 3.2 mm. Each scintillator was coupled to a silicon photomultiplier (SiPM) *via* a wavelength-shifting fibre. We are not aware of any published results of testing of the PRR30 in proton beams although the proof of the technology was successfully demonstrated with smaller prototypes for protons of energy between 100 and 230 MeV.^56,57^

**Figure 8.**
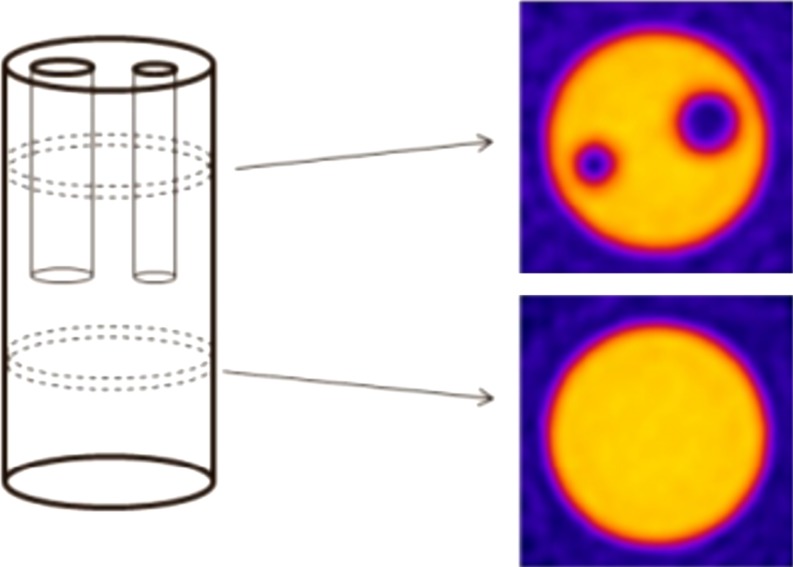
Schematic of a test phantom (left) and two proton CT slices of the phantom obtained with the PRoton IMAging system (right) and published in 2014.^66^ The phantom diameter is 2 cm. © SISSA Medialab Srl. Reproduced from Scaringella et al^66^ with permission from IOP Publishing. All rights reserved.

In 2007, a new pCT group emerged,^60^ although several of the physicists had been previously involved in the early developments for the LLU/UCSC/NIU system.^61^ The new initiative was an Italian project for a PRoton IMAging (PRIMA) device. The general system concept substantially resembled the LLU/UCSC/NIU design: four PSDs based on SiSD technology and a crystal calorimeter as the RERD.^62–64^ However, there were a number of specific differences. The SiSDs used were of a different construction (pitch, 200 μm; thickness, 200 μm); notably the strip thickness was half that of the LLU designs. The RERD was constructed using four YAG : Ce crystals (3 × 3 × 10 cm^3^) arranged in a 2 × 2 array and coupled to photodiodes. A major factor in choice of crystal was the short decay time of YAG : Ce (100 ns)^54^ compared with CsI : Tl (800 ns).^54^ This increased the maximum theoretical proton rate for the calorimeter. The total sensitive area for the first prototype was small at 5.1 × 5.1 cm^2^. The obtained event rate also remained low at 10 kHz. However, characterization has been carried out at both the Laboratori Nazionali del Sud (LNS) (Catania, Italy) with 62-MeV protons and the Svedberg Laboratory (Uppsala, Sweden) with 180-MeV protons.^65^ CT images were reconstructed using the LNS data and example slices of a 2-cm diameter plastic test phantom are reproduced in Figure 8.^67^ Spatial resolution was promising: a full-width half-maximum (FWHM) of 0.9 mm was obtained. However, a low number of acquired projections (every 10^o^), combined with a small phantom and low initial proton energies, makes it difficult to extrapolate image quality to a full-size system.

The PRIMA group has announced the design of their second generation system (PRIMA II).^66^ The sensitive area of the detector will be increased to a more clinically relevant 5 × 20 cm^2^. The larger area is achieved by the use of multiple SiSD in each PSD module. The SiSD thickness has been slightly increased to improve signal-to-noise (320 μm). A larger area for the RERD is achieved by using a higher number of crystals of the same design as PRIMA I but in a 2 × 7 configuration. With a redesigned DAQ, an event rate exceeding 1 MHz is proposed, taking advantage of the fast decay of the YAG : Ce scintillator.

The Particle Residual Energy and Tracker Enhancement project has developed a design based on concepts patented by the Istituto Nazionale di Fisica Nucleare (INFN) (Italy).^68,69^ The pCT system will consist of four PSD modules and a RERD. Each tracker PSD consists of two orthogonal layers of Sci-Fi (BCF-12; decay time, 3.2 ns)^42^ with each fibre having a 0.5 × 05-mm^2^ cross-section. The Sci-Fis are coupled to position-sensitive photomultipliers (PSPMs) *via* clear fibres. The RERD also consists of Sci-Fi technology: in this case, a stack of sixty Sci-Fi layers (BCF-12; 0.5 × 0.5-mm^2^ fibre cross-section). Each Sci-Fi in the RERD is coupled to a PSPM *via* a wave-length shifting fibre. The sensitive area of the initial PSD and RERD prototypes are 20 × 20 and 4 × 4 cm^2^, respectively. A sensitive area of 30 × 30 cm^2^, however, is proposed for the final system. The target event rate is 1 MHz but considerably higher may be possible. Although some parts of the system have been tested in proton beams, radiography and tomography have not yet been presented with the complete system.

NIU, having collaborated with LLU/UCSC in their first generation pCT scanner, has continued with a more local collaboration with the Fermilab National Accelerator Laboratory (FNAL) (Batavia, IL).^70^ The overall concept of the NIU/FNAL scanner bears much in common with the PSI system pioneered in the 1990s. They utilize four PSD tracking units composed of Sci-Fis and a stack of plastic scintillators for the RERD. Each PSD consists of two planes of 0.5-mm diameter polystyrene fibres: these are arranged in triplet bundles for coupling to SiPMs, providing a 0.97-mm detector pitch. Each pair of planes provides a sensitive area of approximately 20 × 24 cm^2^ and has a water-equivalent thickness approaching 2 mm. The RERD consists of a stack of 96 polyvinyltoluene tiles of 3.2-mm thickness. Each tile is 27 × 36 cm^2^ in area and is optically coupled to two SiPMs *via* a wavelength-shifting fibre. The collaboration anticipates imaging an object with a diameter up to 23 cm with a 2-MHz event rate. The scanner is fully assembled and installed for testing at a 200-MeV proton beam facility and initial results can be anticipated in the near future.

Niigata University (Niigata, Japan) has also recently demonstrated a prototype system.^71^ It consists of four PSD units utilizing SiSDs combined with an RERD consisting of a NaI : Tl calorimeter (decay time, 230 ns)^54^ coupled to a photomultiplier tube. The SiSDs provide a 9 × 9 cm^2^ active area (228-μm pitch; 410-μm thickness). Projection radiography has been demonstrated with the system with a 160-MeV beam at a low flux rate of 20 protons cm^−2^s^−1^. The group recognizes that the DAQ is a major limitation of the current system as it permits a maximum acquisition rate of only 30 Hz.

The Proton Radiotherapy Verification and Dosimetry Applications (PRaVDA) consortium, funded by the Wellcome Trust (London, UK), initiated a project to build a pCT and beam monitoring system in early 2013. One of the unique elements of the PRaVDA design is the complete reliance on solid-state devices, rather than scintillator technology. A proof-of-principle has been demonstrated for the use of a range telescope consisting of radiation-hard CMOS APS (the RERD).^41^ Note that unlike a calorimeter or scintillator stack design, where valid measurements require only one proton per scintillator element during a read out cycle, the pixelated nature of a CMOS detector permits many protons to be resolved per frame time. This compensates for the relatively low read-out rate and a proton rate of up to 1 MHz is anticipated. The PSDs will consist of SiSDs (90-μm pitch; 200-μm thickness).^72^ A notable feature of the four tracking PSDs is that each will consist of three SiSD planes (*x*-*u*-*v*) oriented at approximately 120^o^ with respect to each other, rather than the typical two orthogonal planes (*x*-*y*). This will aid the resolution of ambiguities at high proton rates and will be advantageous for monitoring of the beam during treatment.^72^

A summary of the above systems is presented in Table 3. This represents our effort to present a current state of the field. Note, however, that most of the systems are in continued development and also that the summary is not completely exhaustive. For example, a proof-of-principle of a range telescope consisting of multiple layers of nuclear emulsions has been demonstrated at a therapy facility.^73^ Magneto-optics combined with collimation also offers the possibility of conducting pRG by tuning the relationship between object thickness and flux at a distant detector. High spatial resolution images have been demonstrated in such a scheme with relativistic protons (800 MeV).^74^

**Table 3.** A summary of current and recent proton radiography (pRG)/proton CT (pCT) prototypes

Group	Year of reference	Area (cm^2^)	Position-sensitive detector technology (number of units)	Residual energy-range detector technology	Proton rate (Hz)	pCT or pRG
Paul Scherrer Institute^43^	2005	22.0 × 3.2	*x*-*y* Sci-Fi (2)	Plastic scintillator telescope	1 M*^a^*	pRG
LLU/UCSC/NIU^6^	2013	17.4 × 9.0	*x*-*y* SiSDs (4)	CsI (Tl) calorimeters	15 k*^a^*	pCT
LLU/UCSC/CSUSB^55^	2014	36.0 × 9.0	*x*-*y* SiSDs (4)	Plastic scintillator hybrid telescope	2 M*^a^*	pCT
AQUA^59^	2013	30.0 × 30.0	*x*-*y* GEMs (2)	Plastic scintillator telescope	1 M*^a^*	pRG
PRIMA I^66^	2014	5.1 × 5.1	*x*-*y* SiSDs (4)	YAG : Ce calorimeters	10 k*^a^*	pCT
PRIMA II^66^	2014	20.0 × 5.0	*x*-*y* SiSDs (4)	YAG : Ce calorimeters	1 M	pCT
INFN^69^	2014	30 × 30	*x*-*y* Sci-Fi (4)	*x*-*y* Sci-Fi	1 M	pCT
NIU/FNAL^70^	2014	24.0 × 20.0	*x*-*y* Sci-Fi (4)	Plastic scintillator telescope	2 M	pCT
Niigata University^71^	2014	9.0 × 9.0	*x*-*y* SiSDs (4)	NaI(Tl) calorimeter	30*^a^*	pCT
PRaVDA^72^	2015	9.5 × 95	*x-u-v* SiSDs (4)	CMOS APS telescope	1 M	pCT

AQUA, Advanced Quality Assurance; CMOS APS, complementary metal oxide semi-conductor active pixel sensor; CsI : Tl, thallium-doped caesium iodide scintillator; CSUSB, California State University, San Bernadino; INFN, Istituto Nazionale di Fisica Nucleare; FNAL, Fermilab National Accelerator Laboratory; LLU, Loma Linda University; NaI : Tl, thallium-doped sodium iodide scintillator; NIU, Northern Illinois University; PRaVDA, Proton Radiotherapy Verification and Dosimetry Applications; PRIMA, PRoton IMAging; Sci-Fi, scintillating fibre hodoscope; UCSC, University of California Santa Cruz; *x*-*y* (or *x-u-v*) SiSDs, two-plane (or three-plane) silicon strip detectors; YAG : Ce, cerium-doped yttrium aluminium garnet scintillator.

The reference for each system corresponds to the most recent publication for the system in question.

The designation of pCT or pRG indicates whether the initial stated aims include pCT.

^*a*^ Quoted figure (or a value close to it) has been experimentally demonstrated.

## GENERAL DESIGN CONSIDERATIONS

In the previous section, a raft of approaches and technologies were discussed. The acceptability of any design will depend on the relative importance assigned to visual quality (spatial resolution and noise) and quantitative accuracy (fidelity in SPR). The body site being imaged and the environment in which the system will be deployed will also be factors. Given the possible divergences in aims and requirements for which a system may be built, we will limit ourselves to discussing an idealized proton-tracking system and the consequences of some departures from it.

The schematic in Figure 5 illustrates the archetypal design of a pCT/RG system with four PSD modules and an RERD. Table 4 summarizes approximate design constraints for such a system (see the following subsections for further details). The constraints are specified such that the image quality would be limited predominantly by straggling in the patient rather than uncertainties in the measurement of a proton's entry and exit trajectories and residual range. In an ideal system, the weak constraint inequalities would be replaced by strong inequalities (*i.e.* < is replaced with <<). We assume the choice of a range telescope as the RERD and provide a constraint for a calorimeter such that it provides superior performance to an ideal range telescope. A comparison of the theoretical constraints with the design of a real prototype system (LLU/UCSC/NIU) is also presented in Table 4.

**Table 4.** A summary of approximate design constraints for a proton-tracking imaging system. See text for definition of the symbols

Design feature	Constraint value	LLU/UCSC/NIU prototype system
Number of PSDs, *N*	*N* = 4	4
PSD pitch, *P*	$\frac{P}{\sqrt{12}}$ < 1 mm	0.1 mm
PSD offsets, *L*/*D*	$\frac{PL}{\sqrt{6}D}$ < 1 mm	0.5 mm
PSD thickness, *T*	$0.1L\sqrt{\frac{T}{X_{0}}}$ < 1 mm	1.4 mm
RERD discretization, *Δ* (range telescope)	$\frac{\Delta }{\sqrt{12}}$ <3 mm water-equivalent	–
RERD energy resolution, *σ*_E_/*E* (calorimeter)	<0.6% (200 MeV)	0.3% (200 MeV)^50^

LLU, Loma Linda University; NIU, Northern Illinois University; PSD, position-sensitive detector; RERD, residual energy-range detector; UCSC, University of California Santa Cruz.

Calculations for LLU/UCSC/NIU based on: *L*, 150 mm; *D*, 50 mm; *P*, 0.4 mm and *T*, 0.8 mm (Si).^6,52^ Calculations for the RERD are based on initial proton energies of 200 MeV and 1% range straggling.

### Number of position-sensitive detector units

Four is the optimal number of PSD modules, since this number allows reconstruction of both position and direction for the incoming and the outgoing protons. The importance of the first two modules will depend, however, on the proton beam facility. If the beam has a low root mean squared (RMS) spread in proton angles, such as can be assumed for the Gantry 1-beamline at PSI (10 mrad), then the initial proton direction might be reasonably considered well defined.^75^ Furthermore, if the beam is a highly focused spot, as in the original pCT experiments of Hanson et al^21^ (1.6 mm FWHM), then little advantage is gained by having any PSD before the patient. The further reduction of the number of PSD modules to only one after the patient must be considered suboptimal owing to the substantial MCS in the patient (Table 2).

We will not say much regarding proton-integrating designs. We observe, however, that a detector in such a system forms a single PSD after the patient (as part of its function). In this case, it is important to place the imaging receptor as close as possible to the downstream side of the patient, to reduce the blurring effects of patient MCS.

### Spatial resolution of position-sensitive detectors

In an optimal system, the uncertainty on proton path through the system would be limited by MCS in the patient. That is, the spatial resolution of the trackers would be such that the uncertainties on the determined proton positions do not substantially contribute to the overall uncertainty on proton path. The RMS error (RMSE) in reconstructing the proton path inside a patient, owing to MCS within the patient, is of order 1 mm even when using non-linear path estimates.^48^ Based on this figure, a tracker resolution of *σ*_r_ < 1 mm is probably sufficient to consider its contribution sub-dominant. The three main candidate technologies (SiSD, Sci-Fi, GEM) are all based on strip read out in multiple planes. The RMSE in spatial reconstruction with such read out is commonly assumed to come from the discrete width of the strip:

(1) $$\sigma =\frac{P}{\sqrt{12}}$$

where *P* is the strip width.^68^ See Table 4 for the implied design constraint.

### Offsets between position-sensitive detector units

The uncertainty in proton angle in a lateral dimension, based on spatial measurements in two idealized PSDs, can be estimated as:

(2) $$\sigma_{\theta }=\frac{\sqrt{2}}{D}\sigma_{r}=\frac{P}{\sqrt{6}D}$$

where *D* is the separation in PSD modules. This ignores any effects owing to the finite thickness of the PSDs (see Consequences of position-sensitive detector thickness). At the projected distance *L* (see Figure 5), we would therefore require *Lσ*_θ_ < 1 mm to ensure that this effect is sub-dominant. The resulting constraint is presented in Table 4. To control the precision of proton path reconstruction, the distances *L* and *D* must therefore be carefully considered: *L* should be minimized and *D* kept sufficiently large.^52,75–77^ Practical considerations of avoiding collisions of the system with the patient and fitting the system in a treatment room limit the freedom of these choices.

### Consequences of position-sensitive detector thickness

All PSD technologies have a finite detector thickness. The main consequence is a random perturbation in proton direction. This adds to the uncertainty in reconstructing the proton trajectory. The trajectories we want to estimate are those after PSD-2 (immediately before the patient) and before PSD-3 (immediately after the patient). The worst repercussions will be for PSD-3, as the mean proton energy will be lower on exit. The angular dispersion in a thin layer owing to MCS can be estimated using the Rossi–Greisen equation:^78^

(3) $$\sigma_{mcxs}=a\sqrt{\frac{T}{X_{0}}}\;\text{where}\;a=\frac{21.2}{\sqrt{2}}\frac{1}{\beta pc}$$

where *β* is the proton's relativistic speed in units of *c*, *p* is proton momentum, *T* is layer thickness and *X*_*0*_ is the material radiation length. In the energy range of interest, the pre-factor of Equation (3) is: *a* *≈* 0.1. Again, we shall consider an associated projected spatial uncertainty, *Lσ*_*mcs*_< 1 mm, to be sufficiently precise. The resulting constraint is summarized in Table 4.

Note that the SiSD modules in the systems discussed range from approximately 0.5% to 1% of radiation length (0.4–0.8 mm of silicon).^6,66^ The Sci-Fi modules range from approximately 0.25% to 1% of radiation length (1–4 mm of plastic).^33,69^ These numbers were calculated based on elemental radiation lengths^78^ and typical compositions. It has been suggested that GEM detectors typically have a thickness of 1% of radiation length,^59^ which gives comparable scatter.

### Choice of calorimeter, range telescope or hybrid technology

The optimal choice of RERD technology may appear obvious. A calorimeter determines the energy of the outgoing proton and therefore accurately determines its state immediately after the patient. In a range telescope, however, only the stopping depth of the proton is determined. Since there will be statistical variations in penetration depth within the range telescope itself (residual range straggling) this will contribute extra uncertainty on the estimate of WEPL. While this is true, a calorimeter will in fact always possess a finite energy resolution.^50^ In consequence, the superiority of any particular RERD over another cannot be established based on such a general criterion.

Another factor that affects precision of WEPL estimated in a range telescope is the water-equivalent thickness, *Δ*, of the layers of the telescope. Figures in excess of 3 mm, used in some systems, may seem relatively large. However, the uncertainty, *σ*_*Δ*_, owing to discretization is:

(4) $$\sigma_{\Delta }=\frac{\Delta }{\sqrt{12}}$$

where the divisor of $\sqrt{12}$ comes to our aid once more. Consider a beam of protons with an initial range of 26 cm (200 MeV), which, based on a typical straggling slightly in excess of 1%, would exhibit a spread of 3 mm (water-equivalent) in a range telescope. For the discretization uncertainty to be sub-dominant to range straggling, we would require $\Delta /\sqrt{12}$ *<* 3 mm (Table 4).

Calorimeter and range telescope performance can be compared using range-energy relations for protons. For a calorimeter to perform equally well as an ideal telescope for 200-MeV protons (assuming a 1% range uncertainty), an energy resolution of 0.6% would be required (*σ*_E_/*E*). This figure is realistic for a crystal calorimeter.^50^ In any case, two points should be remembered irrespective of RERD technology. Firstly, the uncertainty on initial proton energy will further add to the uncertainty in estimate of WEPL. Secondly, the precision of WEPL determination can be ameliorated by increasing the number of protons in an acquisition. The standard error on an estimate of WEPL for a group of *n* protons will decline with $\sqrt{n}$. Increasing proton number does, however, increase patient imaging dose and scan acquisition time.

It has been suggested that a hybrid technology provides an improvement on a purely calorimeter or range telescope design.^6,55^ By hybrid, we mean: the use of the *signal* in a stack of layers, rather than just where the proton *stops*, to more accurately reconstruct WEPL. This is the approach adopted in the LLU/UCSC/USUSB group. It should be noted that utilizing the amplitude of signal in layers to refine WEPL estimates is also possible for the technologies based on scintillating tiles^57^ and pixel detectors.^41^

### Reconstruction algorithm

The problem of image reconstruction, whether for radiography or tomography, may seem to neatly separate from the problem of technological design. However, images are the final product a system will be judged upon and they depend on the system design in an intimate way. Ideally, reconstruction should be considered simultaneously with technological design. This is especially important owing to the unique problems with reconstruction inherent to this modality. Protons, unlike X-rays, do not follow straight paths in a medium. Strictly, the assumptions of tomography or radiography are violated. However, the deviations from linear paths are commonly mild enough to be considered perturbations. While a scientific literature is being built on the treatment of non-linear paths,^11,21,47,48,79–81^ there is as yet no clear consensus on the optimal reconstruction solution.

A clearly suboptimal approach, however, is to apply strict cuts to reject protons whose paths do not closely conform to linear rays. For example, in initial reconstructions by one group, only 22% of detected protons were accepted.^82^ By adopting this rejection-heavy strategy, the problem becomes conceptually easy. The familiar algorithmic machinery of X-ray CT reconstruction may be used without substantial modification. Conceptual ease comes at the cost of substantially elevated patient dose and acquisition time compared with the rejection-light methods, for the same number of usable protons.

Rejection-light approaches using non-linear path estimates or optimal data-binning strategies are a superior option. Some success has been shown with: an optimal linear ray binning for FBP,^83^ depth-dependent and voxel-specific backprojection for FBP,^84^ a list-mode backprojection-then-filtering algorithm^85^ and iterative reconstruction.^47,51^ As is thematic with tomographic reconstruction, iterative reconstruction provides the most power and flexibility, at the cost of complexity and added computational demands. While reconstruction based on proton-integrating systems might necessitate computational times no greater than conventional X-ray CBCT, in list-mode (proton tracking) algorithms the computational demands are substantially raised. Even so, with appropriate parallelization, it has been shown that even list-mode iterative reconstruction is possible in under 8 min with current technology.^86^ pCT is therefore now feasible for online (near real time) image-guidance and verification in the clinic as well as for off-line planning.

A factor critical to maximizing spatial resolution in tracking systems is the accurate reconstruction of proton paths through the whole system. Inside the patient, protons suffer lateral straggling and follow non-linear paths. Between the patient surface and the adjacent PSDs, however, a proton is assumed to travel in a straight line. Accurate path reconstruction therefore depends on knowledge of the spatial contours of the patient and the intersection of incoming and outgoing proton linear trajectories with this surface. The closer the adjacent PSDs are to the patient's surface, the higher the spatial resolution of images, due the minimization of blurring owing to angular deflections in the detectors. The information on the patient surface can be obtained from a secondary imaging technique,^87^ conducting an initial crude pCT reconstruction^48^ or hull-detection algorithms.^88^ Inside the patient, then, each proton's path can be estimated with varying degrees of sophistication,^47^ using straight lines, cubic splines or statistical models.

## FUTURE OUTLOOK

Both the LLU/UCSC/CSUSB and NIU/FNAL systems are full-size prototypes suitable for scanning the human head and have progressed to installation in therapy centres. Although the final step to use with patients will have its own set of problems, that goal is firmly within reach. Yet, will pCT/pRG ever see widespread clinical use? The answer to this question is unclear. One practical barrier for many current facilities is that they cannot typically access proton energies much in excess of 230 MeV (33 cm range) which would be necessary for the transmission imaging of many body sites. Another difficulty is that fixed-beam proton facilities are widespread, necessitating rotation of the patient. While this poses no fundamental difficulty, a gantry mounted rotation of a pCT system would be preferred for both patient compliance and patient setup.

These authors believe, however, that some form of widespread pRG is inevitable. Just as it has become standard within photon therapy to have the capability for routine imaging of their treatment beam, it will become standard for proton therapy centres. The progression to pRG is a natural next step. This will permit range verification but also enable the use of pRG for image guidance: a long-recognized potential benefit.^32,89^ The matter of pCT is more speculative. While it is acknowledged that the range uncertainty arising from conventional X-ray CT planning alone needs to be improved upon, other imaging modalities offer possibilities. These range from the less exotic (dual-energy CT)^90^ to the more exotic (interaction vertex imaging).^13^ In the opinion of these authors, however, transmission imaging with proton does have some undeniable advantages over other techniques: the same particle is used to image with as to treat with (albeit at a higher energy) and the contrast mechanism (energy loss) relates closely to the quantity of interest (SPR in the patient).

What then will a future clinical pRG/pCT system look like? The precise technology that will prevail remains unclear, although a proton-tracking system should provide the most accurate images for proton therapy planning. It seems unlikely that proton-integrating pRG/pCT devices can provide a fully adequate solution for planning. However, the technological simplicity of these systems and their utility for at least limited range verification may make them a useful stepping-stone to full proton-tracking imaging in the clinic.

## CONCLUSION

This review has summarized the principles of proton transmission imaging, historical developments, modern prototype systems and design issues. Which of the emerging technologies will prevail remains an open question. However, pRG and tomography have enormous potential to improve proton therapy planning and delivery.

## FUNDING

This work was supported by the Wellcome Trust Translation Award Scheme, grant number 098285.
